# Two-color heterodyne laser interferometry for long-distance stage measurement with correction of uncertainties in measured optical distances

**DOI:** 10.1038/s41598-017-07741-4

**Published:** 2017-08-15

**Authors:** Zhaowu Liu, Wenhao Li, Bayanheshig, Xiaotian Li, Shan Jiang, Ying Song, Qiang Lv

**Affiliations:** 1 Changchun Institute of Optics, Fine Mechanics and Physics, Chinese Academy of Sciences, Changchun, Jilin 130033 China; 2 University of Chinese Academy of Science, Beijing, 101408 China

## Abstract

We designed a new system that eliminates deviations by correcting uncertainty in optical distance measurements in the laser two-color heterodyne interferometer. In simulations, eliminating the uncertainty from the atmosphere, the deviation in the uncertainty of the optical distance was 50 times greater with the two-color method than with the one-color method. Adding a correction arm reduces the deviation caused by the uncertainties in measured optical distances. The uncertainty in the measured path length is reduced to 20 nm over a path length of 1500 mm, giving a relative uncertainty of 1.34 × 10^**−**8^.

## Introduction

Heterodyne laser interferometers are used extensively in stage measurements because they have high resolution, precision, speed, and multiple channels^1–3^. However, one of the main limitations in the repetition of long distance measurements is the turbulence of the atmosphere, which affects the refractive index of the air^4, 5^. Atmospheric parameters such as the temperature, pressure, humidity, and possibly the CO_2_ content are measured to calculate the refractive index using the Edlén equation^6, 7^, However, it is difficult to get an accurate distribution of these parameters along the optical path, especially at long distances and with a moving stage^8^. Nevertheless, the influence of the turbulence of the atmosphere can be corrected using the two-color method proposed by Bender and Owens^9, 10^, which is widely used in optical frequency combs^11–14^ and laser interferometers^15–18^. This method measures a geometric distance, *L*, with both wavelength *λ*_1_ and *λ*_2_, to obtain two optical distances, *L*_*λ*1_ and *L*_*λ*2_, and gives the geometric distance *L* = *L*_*λ*1_ − *A*(*L*_*λ*2_ − *L*_*λ*1_), where *A* is the so-called A-coefficient in two-color method that represents the dispersion relation for air refractive indices at two wavelengths.The conventional two-color method eliminated the influence of the atmosphere, yet simultaneously enlarged the uncertainties in measured optical distances, Δ*L*_*λ*1_ and Δ*L*_*λ*2_, because of the stability of the laser wavelength, thermal expansion and the optical thermal drift^19^. These uncertainties were of the order of 10^−8^. The enlargement factor is the same as *A*, and its value is normally high, for example 50 in our study, and the resolution can degrade to 10^−6^ in the case of two-color method, so the uncertainty correction of the measured optical distances is important.

Studies have shown improvement of the resolution by averaging the number of interference fringes counted^16^, and the resolution were evaluated to be more accurate than 2 × 10^−7^. However, this method is not suitable when using a moving stage because information is lost when performing calculations during motion. In the application of optical frequency combs, a monitored interferometer and a probe interferometer were developed to realize highly accurate measurement of the optical distance. An uncertainty of 8.9 × 10^−8^ was achieved^20^. This method compensated for the phase noise and drifts caused by acousto-optic modulators in the system. The monitored interferometer signal is like the reference signal from a laser source in the heterodyne laser interferometer method but this did not solve the laser wavelength stability problem. The stability problem may not be caused by an optical frequency comb but does have serious effects on the heterodyne laser interferometer method.

In this paper, we report a combination of the two-color method and the heterodyne laser interferometer, which can improve the resolution by adding an uncertainty correction system for the measured optical distances when measuring a long distance stage in motion. Using simulations, the influence of the atmosphere and the uncertainty in measured optical distances are successfully eliminated and the uncertainty of the measurement of the 1500 mm distance is less than 20 nm.

## Results

### Theory

**Figure 1:**
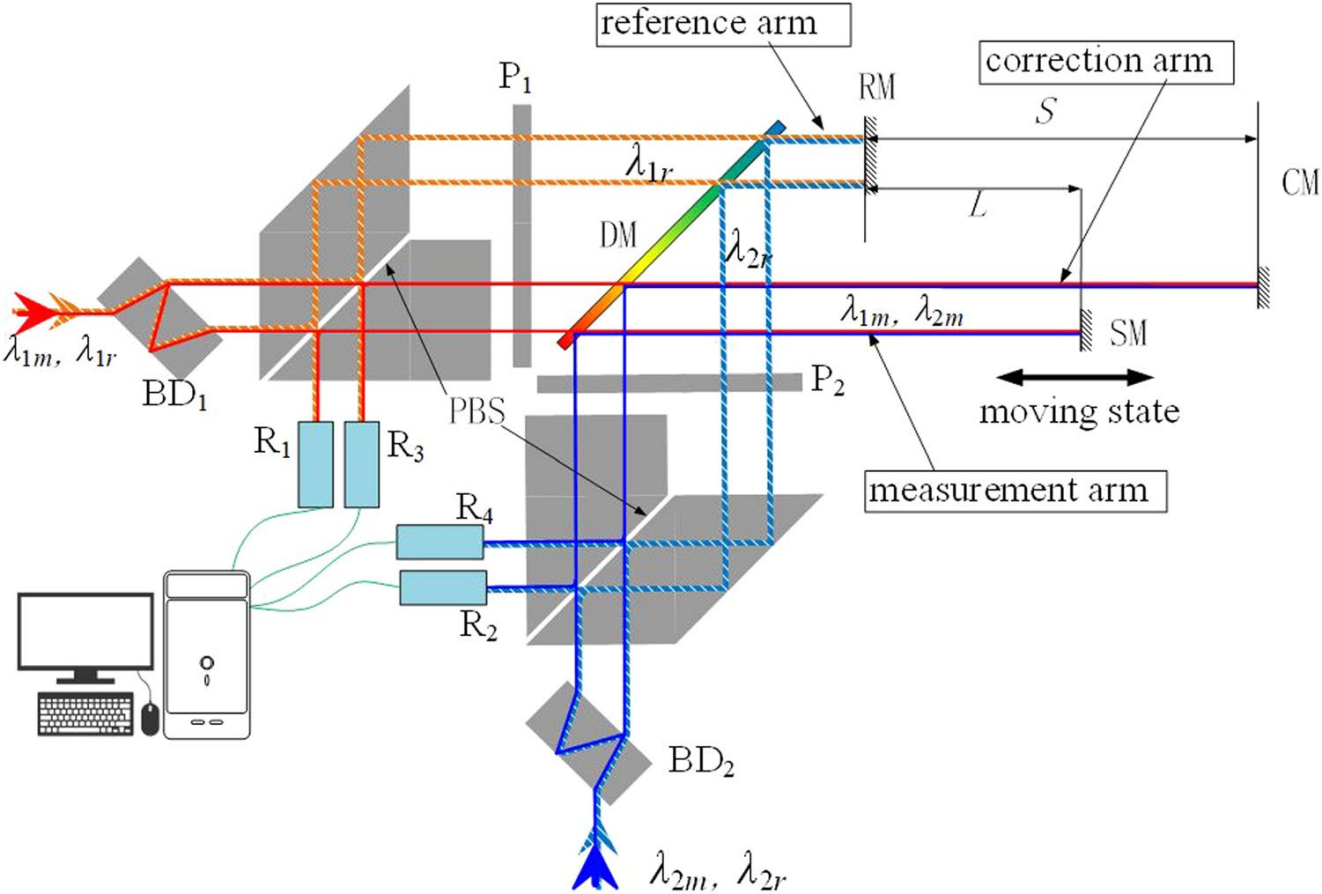
Schematic illustration of the two-color heterodyne laser interferometer with the uncertainty correction of the optical distances. BD_1-2_: beam divider, PBS: polarizing beam-splitter, P_1-2_: quarter-wave plates, DM: dichroic mirror, RM: reference mirror, CM: correction mirror, SM: stage mirror, R_1_~R_4_: Receivers.

A schematic of the two-color heterodyne laser interferometer with a correction of uncertainties in measured optical distances is shown in Fig. 1, both of the two-colored light beams consist of two coherent, collimated, orthogonally polarized frequency components. One is the measurement beam, *λ*_*im*_, and the other is the reference beam, which has a frequency that shifts from *λ*_*im*_ to several MHz and is given by *λ*_*ir*_ (*i* = 1 or 2). Both *λ*_*im*_ and *λ*_*ir*_ are split into two parallel beams using a beam divider. One beam contributes to the measurement and the other is for correction. A polarizing beam splitter reflects *λ*_*ir*_ to the reference arm and transmits *λ*_*im*_ to the measurement arm and the correction arm separately. The return path is superimposed on the outgoing path. Since all beams leaving the polarizing beam splitter pass through a quarter-wave plate, the returning polarizations are rotated by 90°. This causes *λ*_*ir*_ to be transmitted and *λ*_*im*_ to be reflected, so they are directed coaxially to the receivers. The reference path includes two 90° bends so that the reference beams are parallel to their measurement beams or correction beams. The measurement, correction and reference beam paths have the same optical path length in glass. This reduces measurement errors because of changes in temperature.

To keep the same environment, the two color measurement paths are combined with a dichroic mirror to ensure that they have the same optical path. The reference mirror and the correction mirror are fixed to a baseplate made of “0” grade Zerodur with a thermal expansion coefficient below 10^−8^/K between 0 °C and 50 °C. This makes the length of the correction arm to be constant, causing less thermal expansion. The measurement arm and the correction arm are placed as close as possible to the atmosphere to keep their characteristics the same. Several temperature and humidity sensors are set up in the measurement and correction arms to help compensate.

The heterodyne signal of the measurement and reference arm for *λ*_1_ is detected by R_1_ and the heterodyne signal of the measurement and reference arm for *λ*_2_ is detected by R_2_. Combining their reference signals from the laser source, two optical distances, *L*_*λ*1*m*_ and *L*_*λ*2*m*_, can be obtained. The values of *L*_*λ*1*m*_ and *L*_*λ*2m_ are equal to *n*_*λ*1*m*_ *L* and *n*_*λ*2*m*_ *L*, respectively, where *n*_*λ*1*m*_ and *n*_*λ*2*m*_ are the refractive indices of the air at the two wavelengths of the measurement arm. According the theory of the two-color method^17^, the geometric displacement is:

1 $${L}_{m}=\frac{{L}_{\lambda 1m}K({\lambda }_{2})-{L}_{\lambda 2m}K({\lambda }_{1})}{K({\lambda }_{2})-K({\lambda }_{1})-{p}_{wm}[K({\lambda }_{2})\cdot g({\lambda }_{1})-K({\lambda }_{1})\cdot g({\lambda }_{2})]}$$

where *K*(*λ*_*i*_), *g*(*λ*_*i*_) (*i* = 1 or 2) are functions of the wavelength, and *p*_*wm*_ is the pressure of water vapor in the measurement arm. As shown in Equation (1), the dependence on temperature, pressure and CO_2_ content were canceled and only the dependence on the water vapor pressure remained. The change in pressure of the water vapor was calculated by measuring the change in temperature and humidity:

2 $$\begin{array}{rcl}{p}_{w}/{\rm{Pa}} & = & \phi [1.2378847\times {10}^{-5}{(T/{\rm{K}})}^{2}-0.019121316(T/{\rm{K}})\\  &  & +33.93711047-6343.1645/(T/{\rm{K}})]\end{array}$$

where *φ* is the relative humidity, *T* is the air temperature in Kelvin. The influence of the humidity and the temperature remain small and can be measured to compensate for deviation from the water vapor pressure as shown in Equation (2). Therefore, we set several temperature and humidity sensors in the measurement arm. The repeatability of the temperature measurement is given by Δ_*t*_, repeatability in humidity is given by Δ_*φ*_, and the maximum deviation is given by

3 $${e}_{pw}=\pm \sqrt{{(\frac{\partial {L}_{m}/L}{\partial {p}_{w}}\cdot \frac{\partial {p}_{w}}{\partial t}\cdot {{\rm{\Delta }}}_{t})}^{2}+{(\frac{\partial {L}_{m}/L}{\partial {p}_{w}}\cdot \frac{\partial {p}_{w}}{\partial \phi }\cdot {{\rm{\Delta }}}_{\phi })}^{2}}$$

The repeatability of the temperature measurement is Δ_*t*_ < 0.005 °C, the repeatability of the humidity measurement is Δ_*φ*_ < 1% and the measured center wavelength *λ*_1_ = 0.6328 µm, *λ*_2_ = 0.4131 µm. At these wavelengths, the influence of the atmosphere on the two-color heterodyne laser interferometer gives *e*_*pw*_ of <0.024 ppm, which is very small.

While the two-color method successfully eliminated the influence of the atmosphere, the uncertainty in each optical distance measurement, Δ*L*_*λ*1*m*_ and Δ*L*_*λ*2*m*_, increased. Because we set two 90° bends in the reference path, the reference beams are parallel to the related measurement beams and have the same optical path length in glass. This reduces measurement errors because of temperature changes, which result in thermal expansion and optical thermal drift. Therefore, the principal uncertainty of each optical distance is the error in the laser fluctuations, Δ*L*_*λim*_, given by

4 $${\rm{\Delta }}{L}_{\lambda im}={L}_{\lambda im}\cdot \frac{d{\lambda }_{im}}{{\lambda }_{im}}={L}_{\lambda im}\cdot {e}_{laseri},\,i=1\,{\rm{or}}\,2$$

where *e*_*laseri*_ is the wavelength fluctuation of the laser heads. For the two-color method, the deviation of the measured value caused by the uncertainty in each optical distance measurement is:

5 $${\delta }_{Lm}=\frac{\partial {L}_{m}}{\partial {L}_{\lambda 1m}}\cdot {\rm{\Delta }}{L}_{\lambda 1m}+\frac{\partial {L}_{m}}{\partial {L}_{\lambda 2m}}\cdot {\rm{\Delta }}{L}_{\lambda 2im}=\frac{K({\lambda }_{2})\cdot {L}_{\lambda 1m}\cdot {e}_{laser1}-K({\lambda }_{1})\cdot n{L}_{\lambda {\rm{2m}}}\cdot {e}_{laser2}}{K({\lambda }_{2})-K({\lambda }_{1})-{p}_{wm}[K({\lambda }_{2})\cdot g({\lambda }_{1})-K({\lambda }_{1})\cdot g({\lambda }_{2})]}$$

To eliminate the deviation caused by the uncertainty in each optical distance measurement, we design a new optical measurement system by adding an uncertainty correction system for the optical distance. It is almost the same as the two-color heterodyne laser interferometer but the measurement arm, which here is the correction arm, is a constant length, *S*. The heterodyne signal of the correction arm and reference arm for *λ*_1_ is detected by R_3_ and the heterodyne signal of the correction arm and reference arm for *λ*_2_ is detected by R_4_. Two optical distances of the correction arms, *S*_*λ*1*c*_ and *S*_*λ*2*c*_, are obtained. So, the uncertainty in the optical distance from the fluctuations in the laser wavelength at the two wavelengths, Δ*S*_*λ*1*c*_ and Δ*S*_*λ*2*c*_, is:

6 $${\rm{\Delta }}{S}_{\lambda 1c}={n}_{\lambda 1c}S-{S}_{\lambda 1c}={S}_{\lambda 1c}\cdot {e}_{laser1}$$

7 $${\rm{\Delta }}{S}_{\lambda 2c}={n}_{\lambda 2c}S-{S}_{\lambda 2c}={S}_{\lambda 2c}\cdot {e}_{laser2}$$

where *n*_*λ*1*c*_ and *n*_*λ*2*c*_ are the refractive indices of the air at the two wavelengths of the correction arm. By inverting Equation (6) and Equation (7), the laser fluctuation error is:

8 $${e}_{laser1}=\frac{{n}_{\lambda 1c}S-{S}_{\lambda 1c}}{{S}_{\lambda 1c}}$$

9 $${e}_{laser2}=\frac{{n}_{\lambda 2c}S-{S}_{\lambda 2c}}{{S}_{\lambda 2c}}$$

By combining Equations (1), (5), (8) and (9), the geometric displacement with uncertainty in the optical distances is:

10 $$\begin{array}{rcl}{L}_{c} & = & \frac{K({\lambda }_{2}){L}_{\lambda 1m}(1+\frac{{n}_{\lambda 1c}\cdot S-{S}_{\lambda 1c}}{{S}_{\lambda 1c}})-K({\lambda }_{1}){L}_{\lambda 2m}(1+\frac{{n}_{\lambda 2c}\cdot S-{S}_{\lambda 2c}}{{S}_{\lambda 2c}})}{K({\lambda }_{2})-K({\lambda }_{1})-{p}_{wm}[K({\lambda }_{2})\cdot g({\lambda }_{1})-K({\lambda }_{1})\cdot g({\lambda }_{2})]}\\  & = & \frac{K({\lambda }_{2})\frac{{L}_{\lambda 1m}}{{S}_{\lambda 1c}}\cdot {n}_{\lambda 1c}S-K({\lambda }_{1})\frac{{L}_{\lambda 2m}}{{S}_{\lambda 2c}}\cdot {n}_{\lambda 2c}S}{K({\lambda }_{2})-K({\lambda }_{1})-{p}_{wm}[K({\lambda }_{2})\cdot g({\lambda }_{1})-K({\lambda }_{1})\cdot g({\lambda }_{2})]}\end{array}$$

According to the modified Edlén equation^7, 17^, the refractive index of the air is:

11 $${n}_{\lambda ic}-1=K({\lambda }_{i})\cdot D({t}_{c},{p}_{c},{x}_{c})-{p}_{wc}\cdot g({\lambda }_{i}),i=0\,{\rm{or}}\,{\rm{1}}$$

where *t*_*c*_, *p*_*c*_, *x*_*c*_, and *p*_*wc*_ represent the temperature, pressure, CO_2_ concentration and the pressure of water vapor in the correction arm, respectively. Substituting *n*_*λic*_ from Equation (11) into Equation (10), the result is approximately:

12 $$\frac{{L}_{\lambda 1m}}{{S}_{\lambda 1c}}\approx \frac{{L}_{\lambda 2m}}{{S}_{\lambda 2c}}\approx \frac{{L}_{\lambda 1m}-{L}_{\lambda 2m}}{{S}_{\lambda 1c}-{S}_{\lambda 2c}}$$

Equation (10) can rewrite as:

13 $${L}_{c}=S\cdot \frac{\frac{K({\lambda }_{2}){L}_{\lambda 1m}-K({\lambda }_{1}){L}_{\lambda 2m}}{K({\lambda }_{2})-K({\lambda }_{1})-{p}_{wm}[K({\lambda }_{2})\cdot g({\lambda }_{1})-K({\lambda }_{1})\cdot g({\lambda }_{2})]}}{\frac{K({\lambda }_{2}){S}_{\lambda 1c}-K({\lambda }_{1}){S}_{\lambda 2c}}{K({\lambda }_{2})-K({\lambda }_{1})-{p}_{wc}[K({\lambda }_{2})\cdot g({\lambda }_{1})-K({\lambda }_{1})\cdot g({\lambda }_{2})]}}=S\cdot \frac{{L}_{m}}{{S}_{m}}$$

Set *S* be the overall length of the moving stage. The correction mirror is fixed at the terminal position of the stage, which is at the start of the reference mirror. This is beneficial because as the stage moves away from the reference mirror and *L*_*c*_ increases, the effect of the humidity becomes more significant. However, as the path of the measurement arm gets closer to the correction arm, the effect of the humidity on the correction arm can help correct the effect of the humidity on the measurement arm.

### Simulation

In normal laboratory conditions, the temperature *t*_0_ is 20 C, the pressure *p*_0_ is 101325 Pa, the humidity *φ*_0_ is 50%, and the CO_2_ content x_0_ is 0.039%. Using Equation (2), the pressure of the water vapor, *p*_*w*0_, is calculated to be 1170 Pa. The changes in the characteristics of the air in laboratory conditions are listed Table 1. The temperature change consists of two parts. One is the direct influence of the constant, *p*_*w*_, and the other is the indirect influence of *p*_*w*_, with the constant *φ*.

**Table 1:** The disturbance of air parameters and its influence to measurement.

	disturbance of air parameters $d{\rm{\Lambda }}$	
temperature	±0.01 °C	
pressure	±1 HPa	
CO_2_ content	±1 ppm	
pressure of water vapor	temperature	±0.01 °C
	humidity	±10%

**Figure 2:**
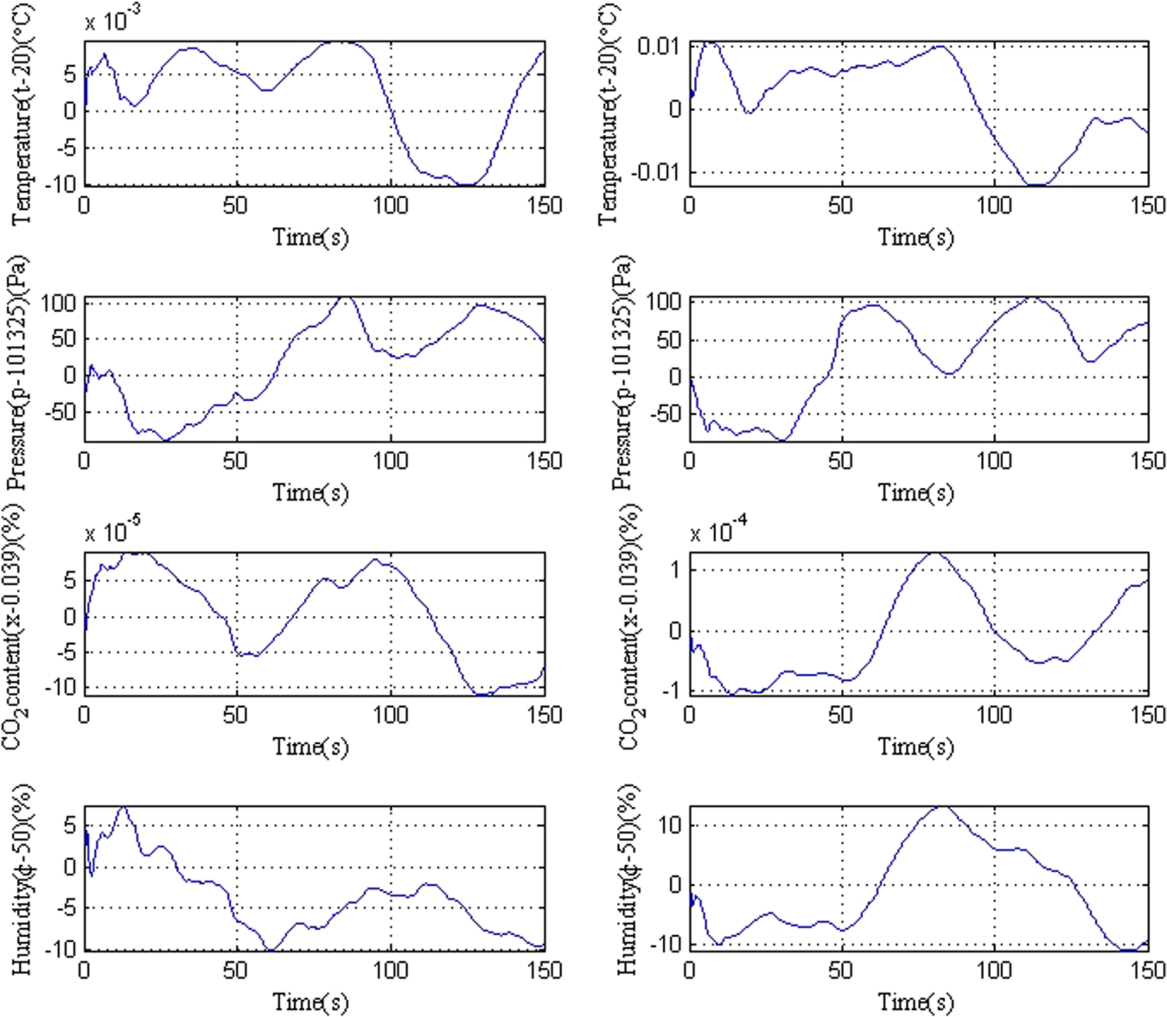
The change in the characteristics of the air: (**a**) the change in the measurement using the left optical path before the stage mirror; (**b**) the change in the measurement using the right optical path after the stage mirror.

To simulate the accuracy of the two-color heterodyne laser interferometer with an uncertainty correction for the measured optical distance, it is assumed that the stage moves from 0 to 1500 mm with a velocity of 10 mm/s. The change in the atmospheric characteristics is generated randomly based on Table I and are shown in Fig. 2. Figure 2(a) displays the disturbance of the left optical path measurement before the stage mirror. Figure 2(b) shows the disturbance of the right optical path after the stage mirror. In 150 s, the change in the characteristics of the air is larger than reality and the difference between the left and right paths is also larger.

**Figure 3:**
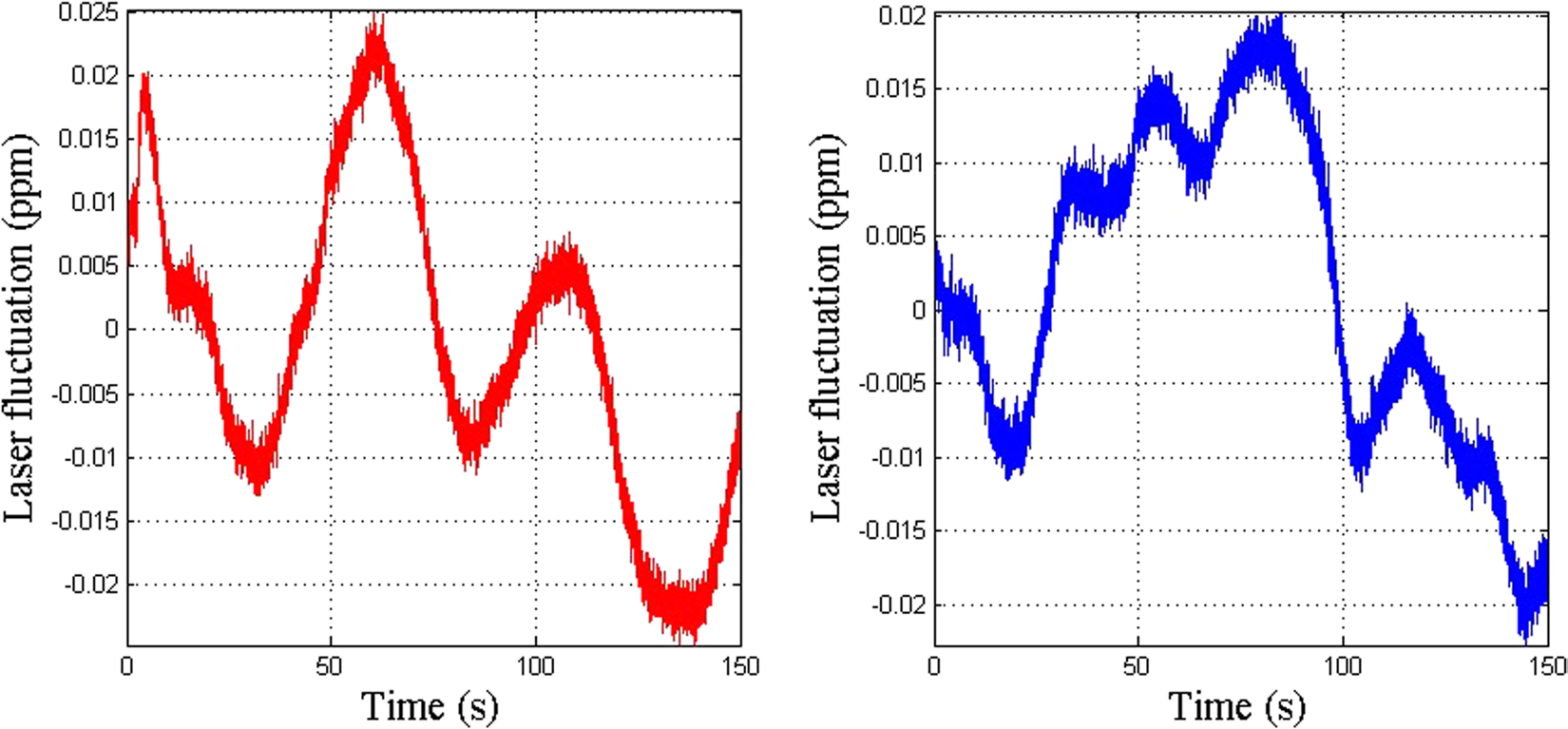
The laser fluctuations: (**a**) the laser fluctuation of the wavelength *λ*_1_ = 0.6328 µm; (**b**) the laser fluctuation of the wavelength *λ*_2_ = 0.4131 µm.

The stability of the wavelength of the laser sources is typically ±0.02 ppm in long-term measurements and ±0.002 ppm in short term measurements. The laser fluctuations generated randomly are shown in Fig. 3. Where (a) is the red laser fluctuation, *e*_*laser*1_, with wavelength *λ*_1_ = 0.6328 µm and (b) is the blue laser fluctuation, *e*_*laser*2_, with wavelength *λ*_2_ = 0.4131 µm.

**Figure 4:**
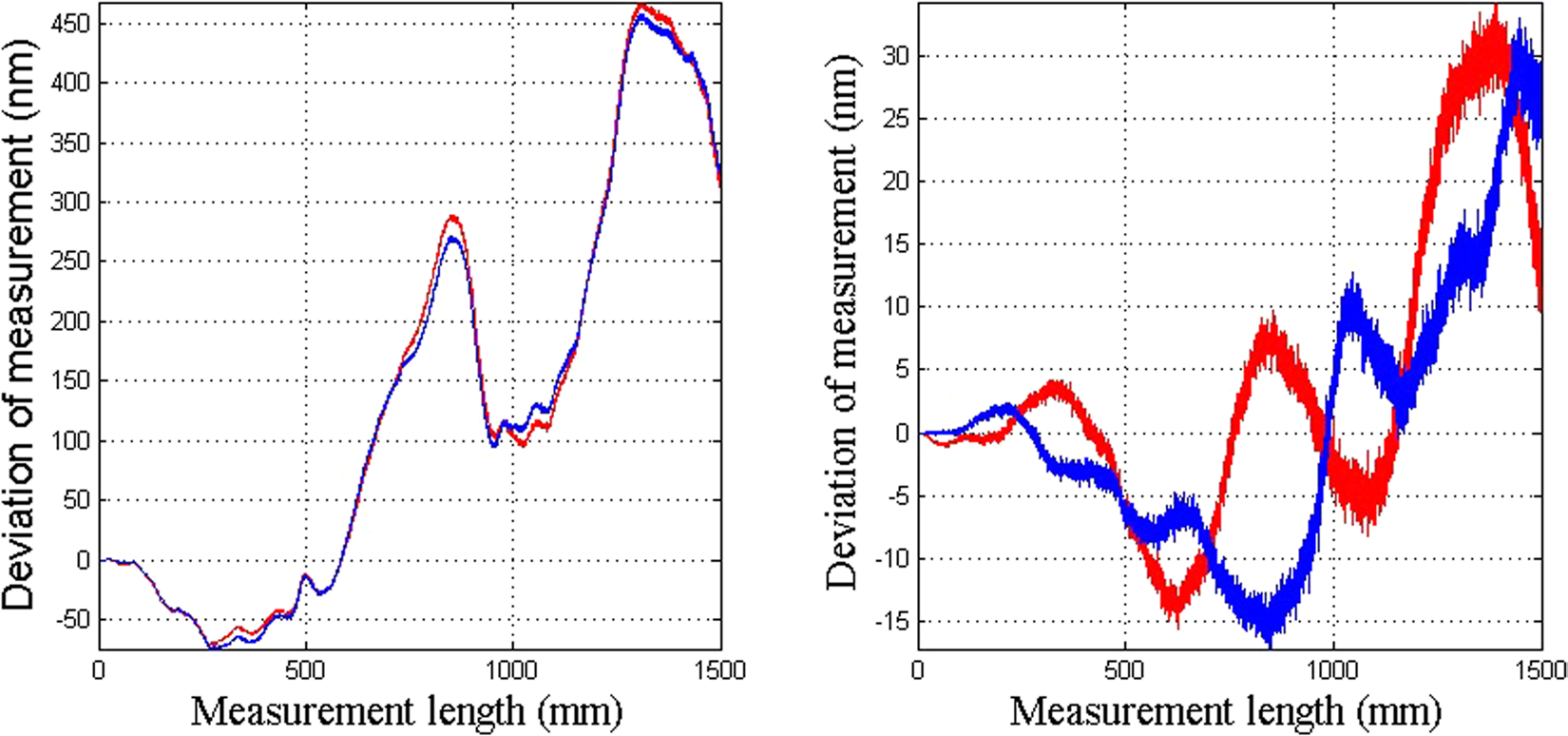
The accuracy of the one-color method: (**a**) the deviation using the one-color method; (**b**) the uncertainties in the optical distance.

The accuracy of the one-color method is shown in Fig. 4(a). The red line is the deviation of the measurement using the red laser *L*_*λ*1*m*_ and the blue line is the deviation of the measurement using the blue laser *L*_*λ*2*m*_. Generally, the deviation of the two colors is roughly identical because the deviations are caused mostly by disturbance of the air, and the two color measurement paths are combined to keep the environment the same. The measurement paths increase as the distance increases. The biggest deviation is 450 nm, which is 3 × 10^−7^ of the measured path length of 1500 mm.

The deviations in the measurement of the two colors are not identical in this case. This is caused by the uncertainty in the measured optical distance. This will also cause uncertainty in the measurement using the two-color method. Figure 4(b) shows the uncertainty in each optical distance measurement, *L*_*λ*1*m*_ and *L*_*λ*2*m*_, separate from Fig. 4(a). The red line is the uncertainty in the optical distance measurement using the red laser and the blue line is the uncertainty in the optical distance measurement using the blue laser. The uncertainties are mostly caused by the change in the wavelength. The biggest deviation is 30 nm, which is 2 × 10^−8^ of the measured path length of 1500 mm.

**Figure 5:**
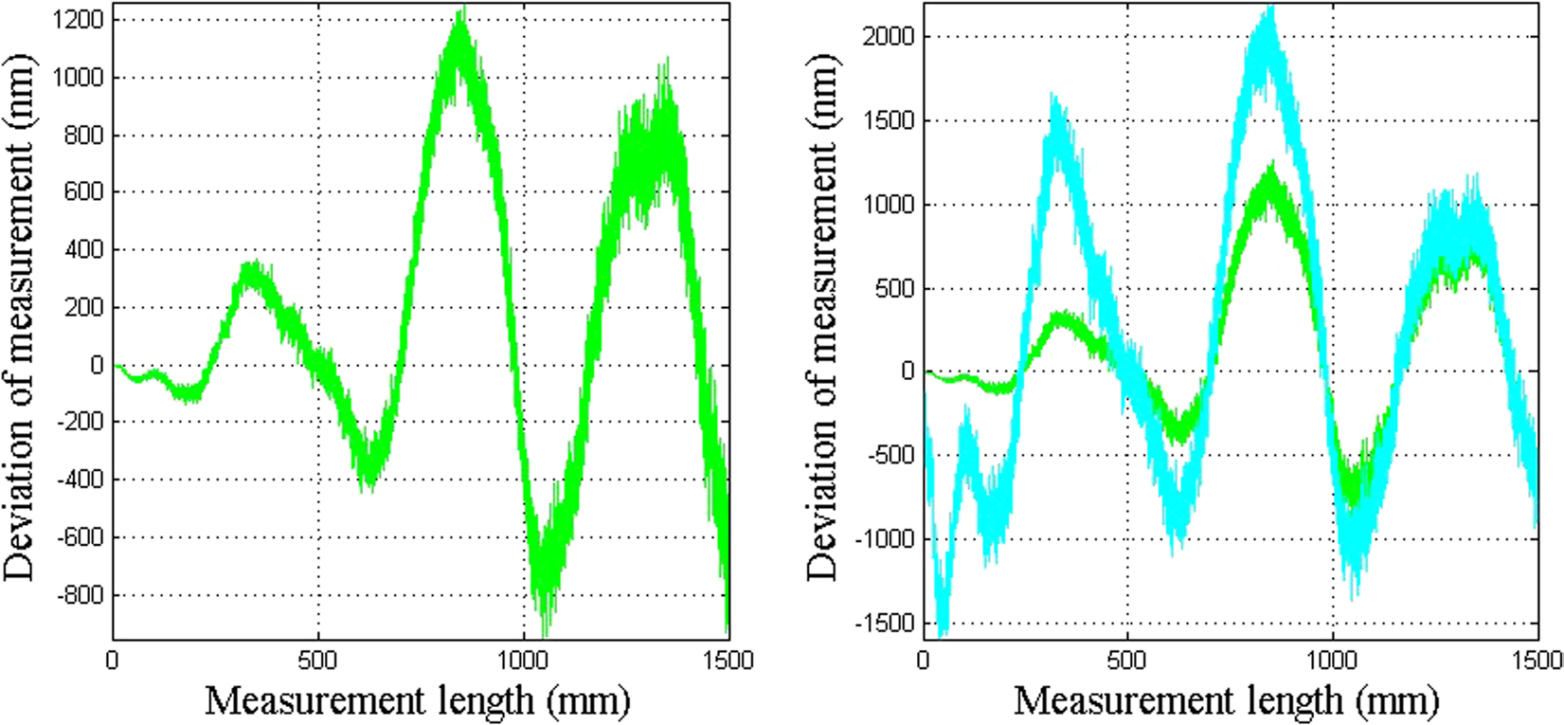
The deviation caused by the uncertainty in each optical distance measurement using the two-color method: (**a**) the deviation of the measurement arm; (**b**) comparison of the deviation in the measurement arm and the correction arm.

Figure 5(a) shows the deviation caused by the uncertainties in measured optical distances. For the two-color method, the biggest deviation is 1200 nm, which is 0.8 × 10^−6^ of the measured path length of 1500 mm. So, even if each optical measurement error is small, their influence on the two-color method cannot be underestimated.

Assuming that the correction arm length is equal to the overall length of the stage (*S* = 1500 mm), Fig. 5(b) shows the comparison of the deviation of the measurement arm and the correction arm. The green line gives the deviation of the measurement arm and the cyan line gives the deviation of the correction arm. The ratio of the two measurement errors is equal to the ratio of the two lengths of the two arms. With the increase in distance, the deviations become close in value.

**Figure 6:**
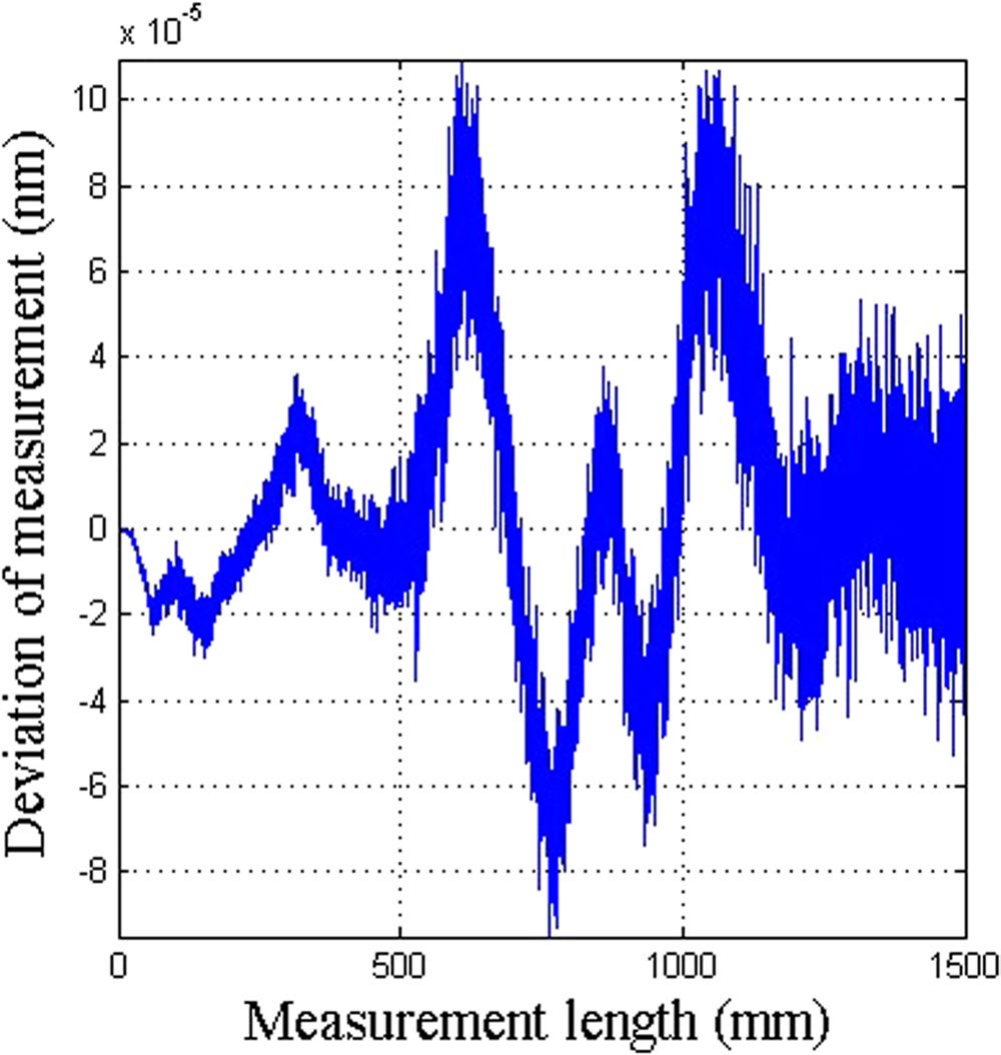
The accuracy of the two-color heterodyne laser interferometer with correction of the uncertainty in the optical distances without considering the uncertainty in the humidity.

The effect of the uncertainty in the humidity is not considered. The geometric distance is calculated using Equation (13), and the measurement error is shown in Fig. 6. The deviation is of the order of 10^−4^ nm, which is 10^−13^ of the measured path length of 1500 mm. This deviation is negligible. This demonstrates that adding the correction arm can correct the deviation from the uncertainties in measured optical distances in the presence of environmental disturbances that have been eliminated by the two-color method.

**Figure 7:**
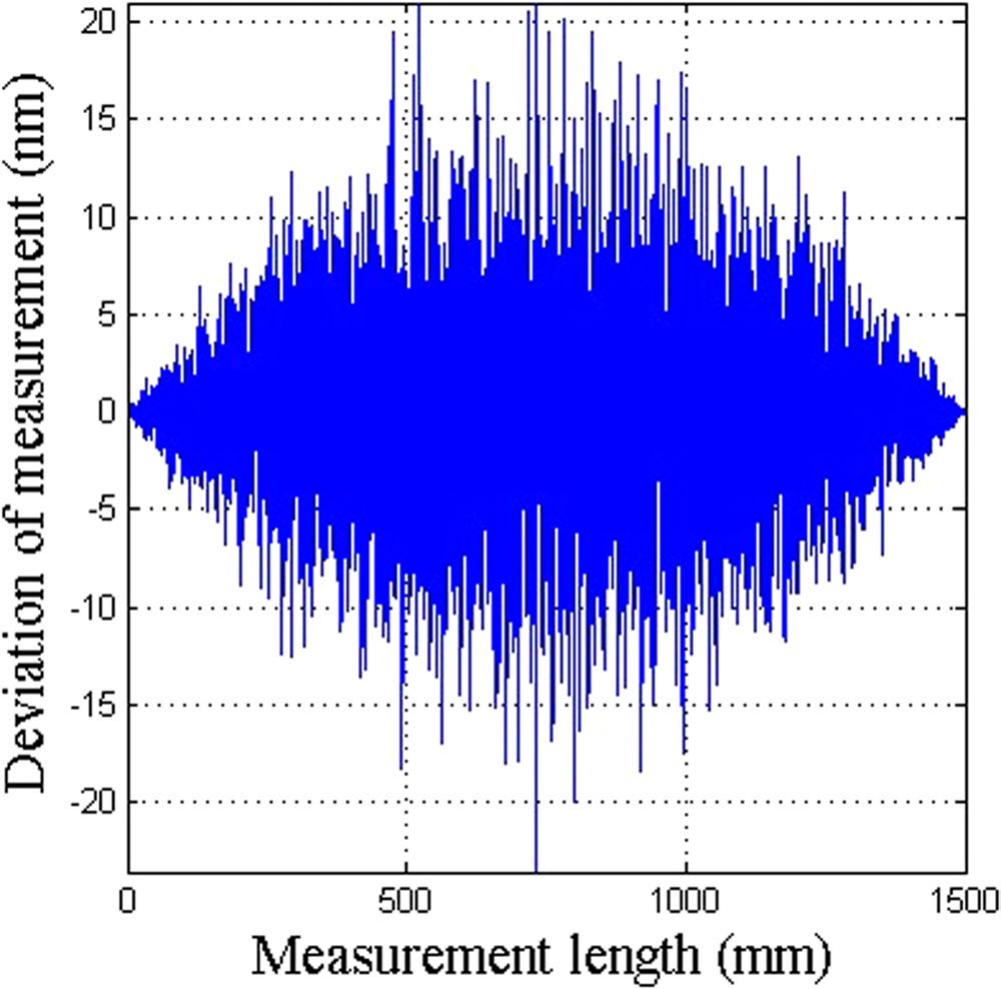
The accuracy of the two-color heterodyne laser interferometer with the correction of the uncertainty in the optical distances with consideration of the uncertainty in the humidity.

In the actual measurements, the accuracy of the humidity will have an impact. Figure 7 shows the accuracy of the two-color heterodyne laser interferometer with correction of the uncertainties in measured optical distances, considering the uncertainty in the humidity measurement as shown in Fig. 2. With the increase in the displacement, the effect of the humidity also increases. When the displacement is more than half of the total displacement, the effect of humidity gradually reduces. This is because we set the correction arm length to be equal to the length of the moving stage. As the stage moves, the path of the measurement arm becomes closer to the correction arm. The effect of the humidity on the correction arm can correct the effect of the humidity on the measurement arm. The biggest deviation is 20 nm, which is 1.34 × 10^−8^ of the measured path length of 1500 mm.

## Discussion

**Figure 8:**
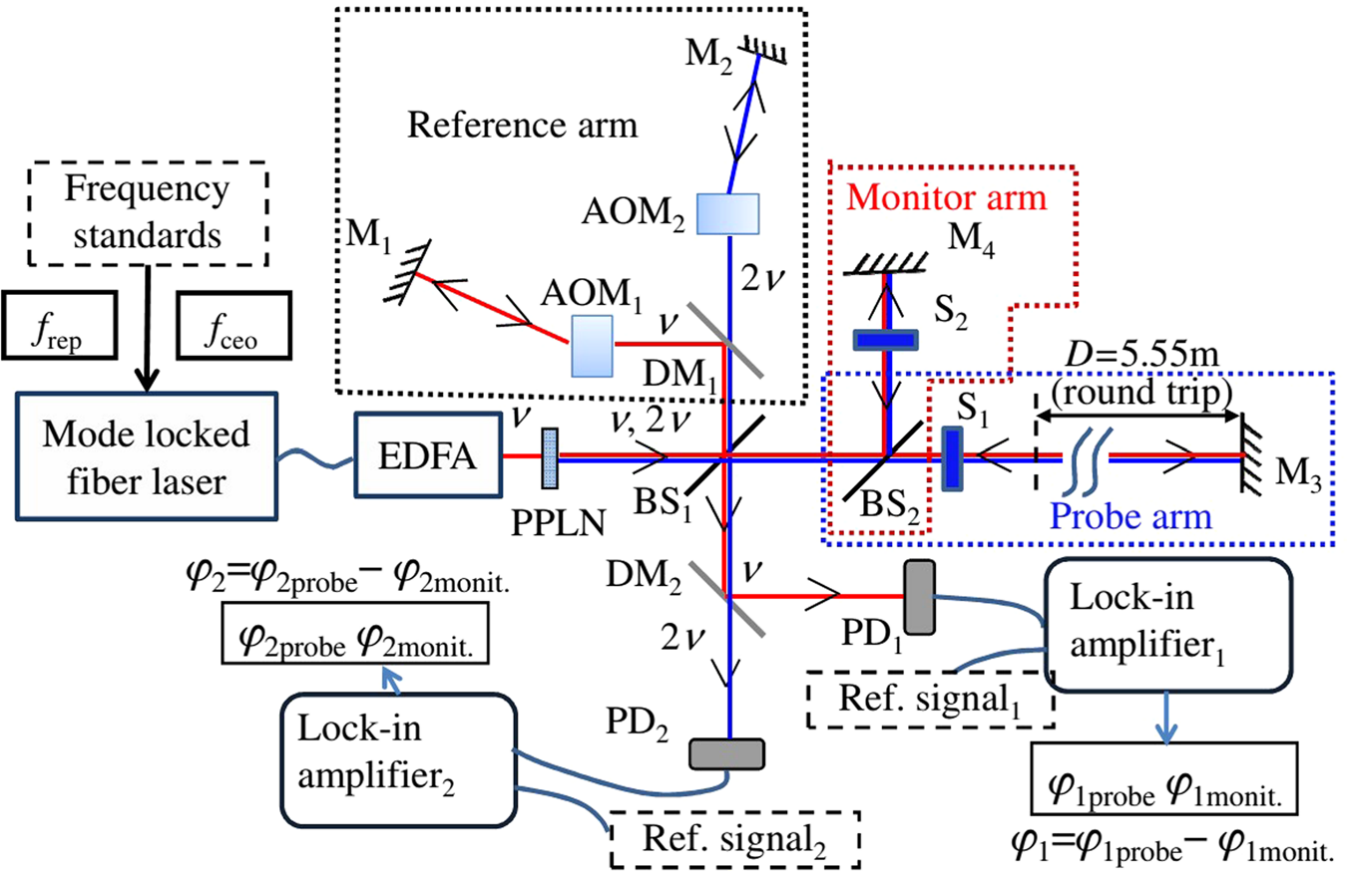
Two-color heterodyne interferometers of optical frequency combs^20^.

Since the two-color method was proposed, it has been used for several decades. In the field of optical frequency comb length measurement^11–14, 20^, Guanhao Wu has published an article in the journal Scientific Reports^12^ and an article in the journal Measurement Science & Technology^20^. Heterodyne pulse-to-pulse interferometers of two-color frequency combs are presented and shown in Fig. 8. In their system, two-color heterodyne pulse to-pulse interferometers based on fundamental and second harmonic generation of the mode-locked laser were developed. Two AOMs were used for the heterodyne signals generation for the two wavelengths. Simultaneously, in order to compensate the phase noise and drift, which were caused by introducing separate reference paths consisting of two AOMs, a monitor interferometer as well as a probe interferometer were constructed in the current system. The variations of optical distances (*L*_1_ and *L*_2_) were precisely obtained by measuring the interferometry phase differences between the probe interferometer and the monitor interferometer. By applying the two-color method, a high accuracy correction of air refractive index with an uncertainty of 8.9 × 10^−8^ was achieved during 10 h continuous measurements while the total refractive index changed with a range of 2.0 × 10^−6^.

This method compensated for the phase noise and drifts caused by AOMs in the system. The monitored interferometer signal is like the reference signal from a laser source in the heterodyne laser interferometer method, but this did not solve the laser wavelength stability problem. The stability problem may not be caused by an optical frequency comb but does have serious effects on the heterodyne laser interferometer method. Anyhow, it proved that the two-color method can eliminate the measurement error caused by the environment. The experimental results are consistent with our simulation results.

**Figure 9:**
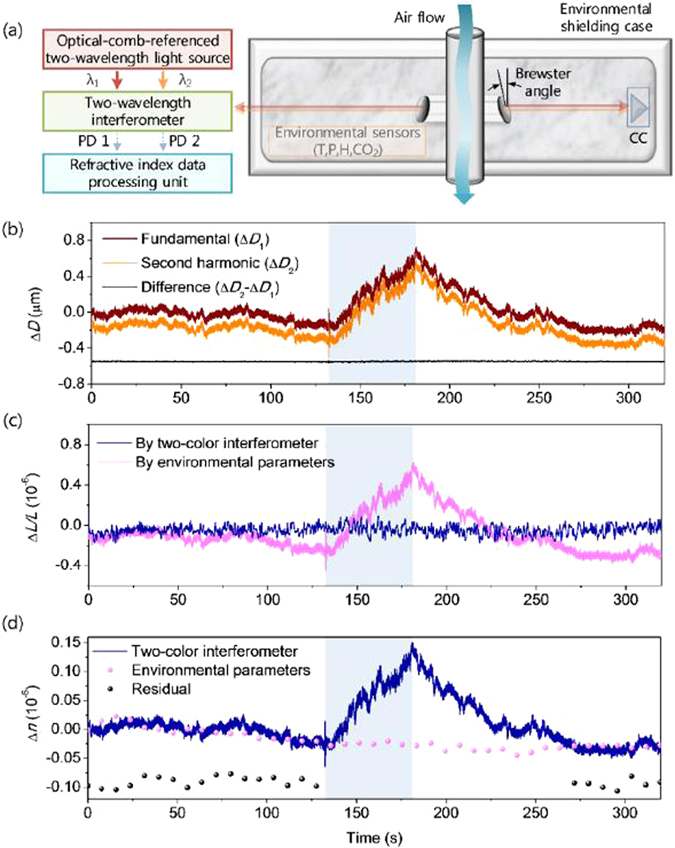
Two-color measurement in dynamic environment^14^.

To more forward a single step, Hyun Jay Kang published an article in the journal Optics Express^14^. They proved the conclusion by partially open the shielding chamber surrounding the interferometer system and the measurement beam line was disturbed by an air jet injected through a nozzle by 2 bar backing pressure during 60 s, shown in Fig. 9(a). The air flow was contained within a glass tube to prevent thermal influence on the granite stage supporting the interferometer. Measured *D*_1_ and *D*_2_ underwent a sudden rise with instability of the order of 10^−6^ as the air jet was turned on as shown in Fig. 9(b). The difference *D*_2_−*D*_1_ was of the order of 10^−9^, being one order of magnitude larger compared to the case of well-controlled environment. Despite the dynamical measurement beam disturbance, the two-color compensation was able to suppress the air refractive index variation to a fractional standard deviation of 4.53 × 10^−8^ throughout the entire measurement period of ~300 s as illustrated in Fig. 9(c). The experimental results also demonstrate that our proposed theoretical simulations are feasible.

The measurement error caused by the environment can be eliminated by the two-color method and has been proved, what we have done is to solve the measurement errors due to uncertainties. In our paper, the compensation arm is inspired by the wavelength tracker of the dual frequency laser interferometer. For Interferometry, the wavelength tracker can be very effective in environmental disturbances and the measurement errors due to uncertainties, but the environmental disturbance is related to the measurement range, for long distances and with a moving stage, the compensation of wavelength tracker for environmental disturbance is limited. The conventional two-color method has already eliminated the influence of the environmental disturbance, measurement errors due to uncertainties is independent of the measurement range, so it can be compensated by the compensation arm.

## Conclusion

We have demonstrated a new optical measurement system to measure the long distance stage while moving. Based on the conventional two-color heterodyne laser interferometerb, this system adds a correction arm to correct the uncertainty of the measured optical distances. Using simulations, we analyzed the performance of the system compared to the one-color heterodyne laser interferometer and to the conventional two-color heterodyne laser interferometer.

The accuracy of the one-color method was 3 × 10^−7^. The deviation of the measurement of the two colors was roughly identical, but was not identical in detail. This caused the uncertainty in the measurement of the optical distance to be 2 × 10^−8^ and the uncertainty in the measurement of the two-color method to be 0.8 × 10^−6^.

Using the new optical measurement system and ignoring the uncertainty in the humidity measurement, the deviation of the order of 10^−4^ nm, which is 10^−13^ of the measured path length of 1500 mm, is negligible. We demonstrate that adding the correction arm can correct the deviation from the uncertainties in the measured optical distances in the presence of environmental disturbances that have been eliminated by the two-color method.

In the actual measurements, when the uncertainty in the humidity is considered, the biggest deviation is 20 nm, which is 1.34 × 10^−8^ of the measured path length of 1500 mm.
